# Supplementary material for: Clinical characteristics, risk factors, and pathogen spectrum of mixed infections in children with Mycoplasma pneumoniae pneumonia: a retrospective cohort study of 1,428 cases from a children’s medical centre in East China
Source: Microbiol Spectr. 2026 May 12;14(6):e03884-25. doi: 10.1128/spectrum.03884-25 (PMC13227972; doi:10.1128/spectrum.03884-25)
Supplement: Supplemental Material — Tables S1 to S6. [file spectrum.03884-25-s0001.docx]

Supplementary materials

Table S1 Comparison of clinical characteristics between single infection group and *M. pneumoniae* mixed bacterial infection group

| Clinical characteristics | Single infection group (n=528) | *M. pneumoniae* mixed bacterial infection group (n=185) | *P* |
| --- | --- | --- | --- |
| Hospital stay (days, IQR) | 9 (7-10) | 8 (7-9.5) | **0.017** |
| Cough duration (days, IQR) | 6( 4-7) | 7 (4-10) | **0.011** |
| Age (years, IQR) | 8 (6-9) | 4 (2-7) | **<0.001** |
| Male, n (%) | 266 (50.38%) | 105 (56.76%) | 0.135 |
| Premature delivery, n (%) | 20 (3.79%) | 12 (6.49%) | 0.128 |
| Vaginal delivery, n (%) | 311 (58.90%) | 122 (65.95%) | 0.091 |
| Severe pneumonia, n (%) | 107 (20.27%) | 71 (38.38%) | **<0.001** |
| History of pneumonia, n (%) | 62 (11.74%) | 31 (16.76%) | 0.081 |
| History of wheezing, n (%) | 31 (5.87%) | 20 (10.81%) | **0.025** |
| Onset season, n (%) |  |  |  |
| Spring | 163 (30.87) | 8 (4.32) | **<0.001** |
| Summer | 171 (32.39) | 62 (33.51) | 0.778 |
| Autumn | 96 (18.18) | 84 (45.41) | **<0.001** |
| Winter | 98 (18.56) | 31 (16.76) | 0.583 |
| Fever grade, n (%) |  |  |  |
| Low fever (<38℃) | 9 (1.70) | 14 (7.57) | **<0.001** |
| Moderate fever (38–39℃) | 156 (29.55) | 60 (32.43) | 0.462 |
| High fever (>39℃) | 346 (65.53) | 89 (48.11) | **<0.001** |
| Symptoms, n (%) |  |  |  |
| Nasal congestion | 141 (26.70) | 70 (37.84) | **0.004** |
| Rhinorrhea | 129 (24.43) | 76 (41.08) | **<0.001** |
| Three Depression Sign | 7 (1.33) | 10 (5.41) | **0.002** |
| Vomiting | 98 (18.56) | 56 (11.09) | **<0.001** |
| Lung signs, n (%) |  |  |  |
| Rhonchi/rales | 33 (6.25) | 10 (5.41) | 0.678 |
| Moist/rales | 422 (79.92) | 126 (68.11) | **0.001** |
| Wheezes/rales | 81 (15.34) | 46 (24.86) | **0.004** |
| Decreased breath sound | 116 (21.97%) | 17 (9.19) | **<0.001** |
| Radiological findings, n (%) |  |  |  |
| Atelectasis | 28 (5.30) | 4 (2.16) | 0.076 |
| Pulmonary consolidation | 50 (9.47) | 7 (3.78) | **0.014** |

Note: Non normal distribution index data (Hospital stay, Cough duration, Age) are represented by median (IQR) and analyzed using *Mann Whitney U tes*t. Other count data are expressed as percentages and analyzed using *χ2* test. The bold column of *P*-values represents meaningful data.

Table S2 Comparison of clinical characteristics between single infection group and *M. pneumoniae* mixed bacterial and viral infection group

| Clinical characteristics | Single infection group (n=528) | *M. pneumoniae* mixed bacterial and viral infection group (n=285) | *P* |
| --- | --- | --- | --- |
| Hospital stay (days, IQR) | 9 (7-10) | 8 (7-9) | **0.002** |
| Cough duration (days, IQR) | 6( 4-7) | 5 (3.5-8) | **<0.001** |
| Age (years, IQR) | 8 (6-9) | 3 (2-5) | **<0.001** |
| Male, n (%) | 266 (50.38%) | 139 (48.77%) | 0.662 |
| Premature delivery, n (%) | 20 (3.79%) | 25 (8.77%) | **0.003** |
| Vaginal delivery, n (%) | 311 (58.90%) | 153 (53.68%) | 0.152 |
| Severe pneumonia, n (%) | 107 (20.27%) | 95 (33.33%) | **<0.001** |
| History of pneumonia, n (%) | 62 (11.74%) | 43 (15.09%) | 0.175 |
| History of wheezing, n (%) | 31 (5.87%) | 44 (15.44%) | **<0.001** |
| Onset season, n (%) |  |  |  |
| Spring | 163 (30.87) | 16 (5.61) | **<0.001** |
| Summer | 171 (32.39) | 104 (36.49) | 0.238 |
| Autumn | 96 (18.18) | 118 (41.40) | **<0.001** |
| Winter | 98 (18.56) | 47 (16.49) | 0.462 |
| Fever grade, n (%) |  |  |  |
| Low fever (<38℃) | 9 (1.70) | 19 (6.67) | **<0.001** |
| Moderate fever (38–39℃) | 156 (29.55) | 83 (29.12) | **0.900** |
| High fever (>39℃) | 346 (65.53) | 137 (48.07) | **<0.001** |
| Symptoms, n (%) |  |  |  |
| Nasal congestion | 141 (26.70) | 175 (61.40) | **<0.001** |
| Rhinorrhea | 129 (24.43) | 201 (70.53) | **<0.001** |
| Three Depression Sign | 7 (1.33) | 22 (7.72) | **<0.001** |
| Vomiting | 98 (18.56) | 102 (35.79) | **<0.001** |
| Lung signs, n (%) |  |  |  |
| Rhonchi/rales | 33 (6.25) | 11 (3.86) | 0.151 |
| Moist/rales | 422 (79.92) | 221 (77.54) | 0.426 |
| Wheezes/rales | 81 (15.34) | 121 (42.46) | **<0.001** |
| Decreased breath sound | 116 (21.97%) | 18 (6.32) | **<0.001** |
| Radiological findings, n (%) |  |  |  |
| Atelectasis | 28 (5.30) | 3 (1.05) | **0.003** |
| Pulmonary consolidation | 50 (9.47) | 3 (1.05) | **<0.001** |

Note: Non normal distribution index data (Hospital stay, Cough duration, Age) are represented by median (IQR) and analyzed using *Mann Whitney U tes*t. Other count data are expressed as percentages and analyzed using *χ2* test. The bold column of *P*-values represents meaningful data.

Table S3 Comparison of clinical characteristics between single infection group and *M. pneumoniae* mixed virus infection group

| Clinical characteristics | Single infection group (n=528) | *M. pneumoniae* mixed virus infection group (n=430) | *P* |
| --- | --- | --- | --- |
| Hospital stay (days, IQR) | 9 (7-10) | 8 (7-9) | **<0.001** |
| Cough duration (days, IQR) | 6( 4-7) | 6 (5-9) | 0.836 |
| Age (years, IQR) | 8 (6-9) | 6 (3-8) | **<0.001** |
| Male, n (%) | 266 (50.38%) | 235 (43.76%) | 0.188 |
| Premature delivery, n (%) | 20 (3.79%) | 14 (3.26%) | 0.654 |
| Vaginal delivery, n (%) | 311 (58.90%) | 270 (50.28%) | 0.220 |
| Severe pneumonia, n (%) | 107 (20.27%) | 108 (25.17%) | 0.073 |
| History of pneumonia, n (%) | 62 (11.74%) | 69 (12.85%) | 0.054 |
| History of wheezing, n (%) | 31 (5.87%) | 29 (5.40%) | 0.579 |
| Onset season, n (%) |  |  |  |
| Spring | 163 (30.87) | 241 (44.88) | **<0.001** |
| Summer | 171 (32.39) | 83 (15.46) | **<0.001** |
| Autumn | 96 (18.18) | 52 (9.68) | **0.009** |
| Winter | 98 (18.56) | 54 (10.06) | **0.011** |
| Fever grade, n (%) |  |  |  |
| Low fever (<38℃) | 9 (1.70) | 13 (2.42) | 0.175 |
| Moderate fever (38–39℃) | 156 (29.55) | 165 (30.73) | **0.004** |
| High fever (>39℃) | 346 (65.53) | 218 (40.60) | **<0.001** |
| Symptoms, n (%) |  |  |  |
| Nasal congestion | 141 (26.70) | 173 (32.16) | **<0.001** |
| Rhinorrhea | 129 (24.43) | 191 (35.57) | **<0.001** |
| Three Depression Sign | 7 (1.33) | 10 (1.86) | 0.244 |
| Vomiting | 98 (18.56) | 97 (18.06) | 0.126 |
| Lung signs, n (%) |  |  |  |
| Rhonchi/rales | 33 (6.25) | 27 (5.03) | 0.985 |
| Moist/rales | 422 (79.92) | 330 (61.45) | 0.233 |
| Wheezes/rales | 81 (15.34) | 104 (19.37) | **<0.001** |
| Decreased breath sound | 116 (21.97%) | 72 (13.41) | **0.043** |
| Radiological findings, n (%) |  |  |  |
| Atelectasis | 28 (5.30) | 15 (2.80) | 0.177 |
| Pulmonary consolidation | 50 (9.47) | 28 (5.21) | 0.096 |

Note: Non normal distribution index data (Hospital stay, Cough duration, Age) are represented by median (IQR) and analyzed using *Mann Whitney U tes*t. Other count data are expressed as percentages and analyzed using *χ2* test. The bold column of *P*-values represents meaningful data.

Table S4 Comparison of clinical characteristics between single infection group and *M. pneumoniae* mixed bacterial infection group

| Lab test | Single infection group (n=528) | *M. pneumoniae* mixed bacterial infection group (n=185) | *P* |
| --- | --- | --- | --- |
| C3 (g/L) | 1.28 (1.14-1.42) | 1.27 (1.14-1.40) | 0.358 |
| C4 (g/L) | 0.42±0.12 | 0.39±0.13 | **0.004** |
| IgA (g/L) | 1.40±0.67 | 1.10±0.68 | **<0.001** |
| IgG (g/L) | 9.18 (7.72-10.90) | 8.77 (7.11-10.23) | **0.008** |
| IgM (g/L) | 1.19 (0.93-1.48) | 1.27 (0.91-1.65) | 0.209 |
| CD3+ (%) | 67.76±8.19 | 67.85±9.00 | 0.915 |
| CD3+CD4+ (%) | 36.65±8.19 | 38.31±8.49 | **0.037** |
| CD3+CD8+ (%) | 25.38±6.00 | 24.31±6.60 | 0.07 |
| CD4+/CD8+ (%) | 1.48 (1.13-1.88) | 1.60 （1.19-2.09） | **0.031** |
| CD3-CD19+ (%) | 19.03±6.79 | 19.82±7.84 | 0.245 |
| CD3-CD(16+56)+(%) | 10.89 (6.76-16.33) | 10.51 （6.58-14.66） | 0.189 |
| CD19+CD23+ (%) | 5.80 (4.03-8.59) | 5.75 （3.95-8.62） | 0.696 |
| PT (s) | 13.48±0.77 | 13.34±0.95 | 0.077 |
| APTT (s) | 39.37±5.69 | 39.56±5.63 | 0.714 |
| Fib (g/L) | 4.80 (4.20-5.29) | 4.64 (3.95-5.32) | 0.128 |
| TT (s) | 15.83±0.95 | 15.83±1.12 | 0.957 |
| AT-III (%) | 111.35±10.99 | 111.75±12.26 | 0.696 |
| DD-PLUS (μg/L) | 580 (410-840) | 460 (322.5-750) | <**0.001** |
| FDP (μg/L) | 2370 (1792.50-3117.50) | 2330 (1762.50-3182.50) | 0.81 |
| PCT (ng/mL) | 0.11 (0.07-0.15) | 0.10 (0.06-0.36) | 0.468 |
| SAA (mg/L) | 127.10 (54.90-235) | 48.30 (6-213.20) | **0.006** |
| HBP (ng/mL) | 39.72 (18.21-79.73) | 43.97 (17.55-113.96) | 0.539 |
| sCRP (mg/L) | 13.12 (6.35-25.96) | 12.47 (3.45-28.28) | 0.412 |

Note: Non normal distribution index data (C3, IgG, IgM, CD4+/CD8+, CD3-CD (16+56)+, CD19+CD23+, Fib, DD-PLUS, FDP, PCT, SAA, HBP, sCRP) are represented by median (IQR) and analyzed using *Mann Whitney U test*. Other indicators of normal distribution data are expressed as mean ± SD, and *t-test* is used for statistical analysis. The bold column of *P*-values represents meaningful data.

Table S5 Comparison of clinical characteristics between single infection group and *M. pneumoniae* mixed bacterial-viral infection group

| Lab test | Single infection group (n=528) | *M. pneumoniae mixed bacterial-viral infection group (n=285)* | *P* |
| --- | --- | --- | --- |
| C3 (g/L) | 1.28 (1.14-1.42) | 1.16 （1.02-1.35） | **<0.001** |
| C4 (g/L) | 0.42±0.12 | 0.36±0.10 | **<0.001** |
| IgA (g/L) | 1.40±0.67 | 0.81±0.55 | **<0.001** |
| IgG (g/L) | 9.18 (7.72-10.90) | 7.82 （6.21-9.29） | **<0.001** |
| IgM (g/L) | 1.19 (0.93-1.48) | 1.29 （1-1.72） | **0.003** |
| CD3+ (%) | 67.76±8.19 | 64.48±11.05 | **<0.001** |
| CD3+CD4+ (%) | 36.65±8.19 | 37±8.75 | 0.620 |
| CD3+CD8+ (%) | 25.38±6.00 | 23.04±7.54 | **<0.001** |
| CD4+/CD8+ (%) | 1.48 (1.13-1.88) | 1.62 （1.29-2.10） | **<0.001** |
| CD3-CD19+ (%) | 19.03±6.79 | 24.48±9.49 | **<0.001** |
| CD3-CD(16+56)+ (%) | 10.89 (6.76-16.33) | 8.20 (5.70-12.61) | **<0.001** |
| CD19+CD23+ (%) | 5.80 (4.03-8.59) | 7.42 （4.50-11.05） | **<0.001** |
| PT (s) | 13.48±0.77 | 13.21±0.98 | **<0.001** |
| APTT (s) | 39.37±5.69 | 40.57±6.64 | **0.015** |
| Fib (g/L) | 4.80 (4.20-5.29) | 4.23 （3.52-5.17） | **<0.001** |
| TT (s) | 15.83±0.95 | 15.89±1.11 | 0.46 |
| AT-III (%) | 111.35±10.99 | 113.12±13.26 | 0.069 |
| DD-PLUS (μg/L) | 580 (410-840) | 430 （310-560） | **<0.001** |
| FDP (μg/L) | 2370 (1792.50-3117.50) | 2230 （1752.50-2957.50） | 0.255 |
| PCT (ng/mL) | 0.11 (0.07-0.15) | 0.11 (0.07-0.24) | 0.105 |
| SAA (mg/L) | 127.10 (54.90-235) | 76.50 (35.90-183.50) | 0.117 |
| HBP (ng/mL) | 39.72 (18.21-79.73) | 38.96 (18.14-74.21） | 0.803 |
| sCRP (mg/L) | 13.12 (6.35-25.96) | 8.64 （2.56-23.58） | **<0.001** |

Note: Non normal distribution index data (C3, IgG, IgM, CD4+/CD8+, CD3-CD (16+56)+, CD19+CD23+, Fib, DD-PLUS, FDP, PCT, SAA, HBP, sCRP) are represented by median (IQR) and analyzed using *Mann Whitney U test*. Other indicators of normal distribution data are expressed as mean ± SD, and *t-test* is used for statistical analysis. The bold column of *P*-values represents meaningful data.

Table S6 Comparison of clinical characteristics between single infection group and *M. pneumoniae* mixed virus infection group

| Lab test | Single infection group (n=528) | *M. pneumoniae mixed virus infection group (n=430)* | *P* |
| --- | --- | --- | --- |
| C3 (g/L) | 1.28 (1.14-1.42) | 1.26 (1.12-1.38) | **0.042** |
| C4 (g/L) | 0.42±0.12 | 0.38±0.13 | **<0.001** |
| IgA (g/L) | 1.40±0.67 | 1.25±0.71 | **0.001** |
| IgG (g/L) | 9.18 (7.72-10.90) | 8.74 (7.23-10.41) | **0.001** |
| IgM (g/L) | 1.19 (0.93-1.48) | 1.27 (0.95-1.68) | **0.011** |
| CD3+ (%) | 67.76±8.19 | 67.09±9.18 | 0.299 |
| CD3+CD4+ (%) | 36.65±8.19 | 36.80±8.10 | 0.804 |
| CD3+CD8+ (%) | 25.38±6.00 | 25.15±6.88 | 0.625 |
| CD4+/CD8+(%) | 1.48 (1.13-1.88) | 1.49 (1.14-1.87) | 0.577 |
| CD3-CD19+ (%) | 19.03±6.79 | 20.45±8.50 | **0.013** |
| CD3-CD(16+56)+ (%) | 10.89 (6.76-16.33) | 9.80 (6.46-15.05) | 0.066 |
| CD19+CD23+ (%) | 5.80 (4.03-8.59) | 5.93 (3.80-9.40) | 0.712 |
| PT (s) | 13.48±0.77 | 13.28±0.80 | **<0.001** |
| APTT (s) | 39.37±5.69 | 38.72±5.78 | 0.091 |
| Fib (g/L) | 4.80 (4.20-5.29) | 4.49 (3.90-4.99) | **<0.001** |
| TT (s) | 15.83±0.95 | 15.96±1.03 | 0.057 |
| AT-III (%) | 111.35±10.99 | 114.77±11.31 | **<0.001** |
| DD-PLUS (μg/L) | 580 (410-840) | 495 (330-780) | **<0.001** |
| FDP (μg/L) | 2370 (1792.50-3117.50) | 2190 (1665-3027.50） | 0.065 |
| PCT (ng/mL) | 0.11 (0.07-0.15) | 0.09 (0.01-0.14) | **0.003** |
| SAA (mg/L) | 127.10 (54.90-235) | 73.15 (26.78-186.45) | 0.055 |
| HBP (ng/mL) | 39.72 (18.21-79.73) | 41.27 (21.34-67.77) | 0.859 |
| sCRP (mg/L) | 13.12 (6.35-25.96) | 8.34 (3.19-18.23) | **<0.001** |

Note: Non normal distribution index data (C3, IgG, IgM, CD4+/CD8+, CD3-CD (16+56)+, CD19+CD23+, Fib, DD-PLUS, FDP, PCT, SAA, HBP, sCRP) are represented by median (IQR) and analyzed using *Mann Whitney U test*. Other indicators of normal distribution data are expressed as mean ± SD, and *t-test* is used for statistical analysis. The bold column of *P*-values represents meaningful data.
